# Synthesis, stabilization, and characterization of the MR1 ligand precursor 5-amino-6-D-ribitylaminouracil (5-A-RU)

**DOI:** 10.1371/journal.pone.0191837

**Published:** 2018-02-05

**Authors:** Kelin Li, Charles K. Vorkas, Ashutosh Chaudhry, Donielle L. Bell, Richard A. Willis, Alexander Rudensky, John D. Altman, Michael S. Glickman, Jeffrey Aubé

**Affiliations:** 1 Division of Chemical Biology and Medicinal Chemistry, UNC Eshelman School of Pharmacy, University of North Carolina, Chapel Hill, North Carolina, United States of America; 2 Division of Infectious Diseases, Weill Cornell Medicine, New York, New York, United States of America; 3 Immunology Program, Sloan Kettering Institute, New York, New York, United States of America; 4 Department of Microbiology and Immunology, Emory University School of Medicine, Atlanta, Georgia, United States of America; 5 Division of Infectious Diseases, Memorial Sloan Kettering Cancer Center, New York, New York, United States of America; La Trobe University, AUSTRALIA

## Abstract

Mucosal-associated invariant T (MAIT) cells are an abundant class of innate T cells restricted by the MHC I-related molecule MR1. MAIT cells can recognize bacterially-derived metabolic intermediates from the riboflavin pathway presented by MR1 and are postulated to play a role in innate antibacterial immunity through production of cytokines and direct bacterial killing. MR1 tetramers, typically stabilized by the adduct of 5-amino-6-D-ribitylaminouracil (5-A-RU) and methylglyoxal (MeG), are important tools for the study of MAIT cells. A long-standing problem with 5-A-RU is that it is unstable upon storage. Herein we report an efficient synthetic approach to the HCl salt of this ligand, which has improved stability during storage. We also show that synthetic 5-A-RU•HCl produced by this method may be used in protocols for the stimulation of human MAIT cells and production of both human and mouse MR1 tetramers for MAIT cell identification.

## Introduction

The study of mucosal-associated invariant T cells (MAIT cells) represents an intriguing frontier in immunology [1–10]. MAIT cells are prevalent within the human peripheral T cell compartment, gut, lung and liver with large inter-individual variability associated with age and disease states, composing as little as <1% to >10% of peripheral blood CD3+ cells [11–14]. Despite such abundance in human donors, their specific roles during the innate immune response are incompletely understood. MAIT cells canonically express a semi-invariant T-cell receptor (TCR) composed of TRAV1-2-TRAJ33 α-chain pairing predominantly with TRBV6 and TRBV20 β-chains [15–17]. Upon activation, MAIT cells can produce granzyme B, interferon-γ, tumor necrosis factor-α, interleukin-17, and kill bacterially infected cells [11, 18, 19]. There is mounting evidence that MAIT cells may play a key role in the detection and response to infectious pathogens, including *Mycobacterium tuberculosis* (*Mtb*) [12, 20–22].

**Fig 1:**
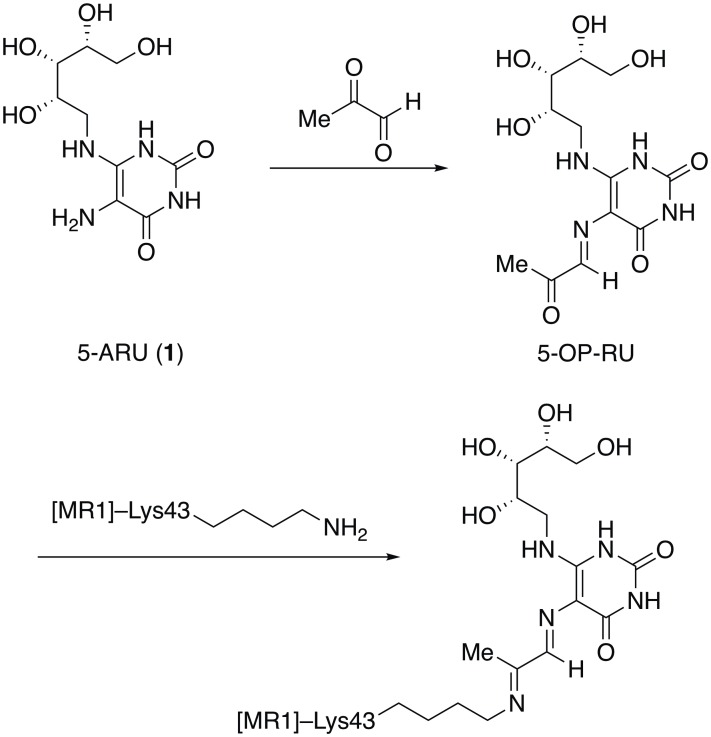
Chemical structures. Structures of 5-A-RU (**1**), the Schiff base it forms with methylglyoxal (5-OP-RU), and the covalent attachment of the latter to MR1.

An enabling advance in MAIT cell biology was the discovery that the MAIT cell TCR recognizes microbially-derived vitamin B metabolites presented by the major histocompatibility (MHC)-related protein MR1 [7, 23]. A number of stabilizing ligands were initially discovered, some of which were reported to activate MAIT cells, while others (notably 6-formylpterin) bound tightly to MR1 but had minimal stimulatory activity [16, 24]. In addition to providing a molecular basis for the detection of MAIT cells, these observations also enabled the construction of antigen-loaded MR1 tetramers, first using reduced 6-hydroxymethyl-8-D-ribityllumazine as a stabilizing ligand [16]. In later work, intermediates for riboflavin biosynthesis such as 5-amino-6-D-ribitylaminouracil (5-A-RU, **1**, Fig 1), modified *in situ* by methylglyoxal (MeG), were discovered to be potent activators of MAIT cells and also allowed generation of MR1 tetramers [24]. In the latter case, Corbett et al. demonstrated that 5-A-RU forms a Schiff base adduct 5-OP-RU with MeG, thus presenting a highly activated ketone to Lys43 of MR1 for covalent attachment, which stabilizes MR1 and permits the formation of stable complexes [24]. The resulting tetramers have proved capable of binding MAIT cells directly and as such have emerged as the gold standard for identification of MAIT cells, replacing less specific approaches such as staining for TRAV1-2 in combination with the C-type lectin receptor, CD161.

5-A-RU has been known for many years due to its role as an intermediate in the biosynthesis of riboflavin [25, 26]. Accordingly, syntheses of 5-A-RU and congeners have been reported since the late 1950s, with key early advances registered by Plaut [27, 28], Katagiri [29], Masuda [30], and Wood [31–33]. These procedures were largely adapted by later investigators [34–37]; in addition, an enzymatic approach has also been reported [38]. Consistently, these investigators have remarked on the difficulty of working with 5-A-RU once it has been synthesized, citing stability problems that necessitated some combination of avoiding light, air, or concentrated solutions. In some cases, these problems were minimized by directly using freshly prepared solutions of 5-A-RU in the intended subsequent reaction, but even this workaround has the disadvantage of needing to prepare **1** on each occasion of use. The specific nature of the instability of **1** has not been established, although it has been described as prone to oxidation [36]. We have confirmed that the material does decompose to unidentified byproducts when left for even a few hours (see below).

The use of **1** as a ligand for MR1 folding reactions requires the *in situ* reaction of **1** with MeG to afford the active species 5-OP-RU. Left standing, this initial Schiff base is known to cyclize to afford the corresponding lumazine [39]. In this situation, having a stable store of 5-A-RU is desirable, but the aforementioned stability problems complicate this approach. An alternative approach, published by Mak et al. when the present manuscript was in preparation, involves the preparation of solutions of 5-OP-RU in DMSO, which were reported to be stable [37].

In this paper, we report that the stability and convenience of **1** can be enhanced through the simple expedient of making and storing it as its HCl salt. In the course of this work, we combined the best features of existing syntheses of **1** into a practical route. Finally, we show that **1**•HCl, when reacted with MeG, performs comparably to 5-OP-RU made by other routes as demonstrated by upregulation of the surface expression of MR1, activation of MAIT cells, and the construction of MR1 tetramers.

## Materials and methods

### Chemistry

The syntheses of (2*R*,3*S*,4*S*)-5-aminopentane-1,2,3,4-tetraol (**2**) [29], 6-chloro-5-nitropyrimidine-2,4(1*H*,3*H*)-dione (**3b**) [31, 40], and 5-nitro-6-(((2*S*,3*S*,4*R*)-2,3,4,5-tetrahydroxypentyl)amino)pyrimidine-2,4(1*H*,3*H*)-dione (**4c**) [31] were carried out by previously reported routes. They are included in Supporting Information S1 File along with full characterization of the intermediates.

### 5-Amino-6-D-ribitylaminouracil hydrochloride (1•HCl, 5-A-RU•HCl)

To a solution of 5-nitro-6-(((2*S*,3*S*,4*R*)-2,3,4,5-tetrahydroxypentyl)amino)-pyrimidine-2,4(1H,3H)-dione (**4c**, 0.327 mmol, 100 mg) in water (4 mL) was added 2 drops of 2N aqueous KOH followed by sodium hydrosulfite (1.96 mmol, 0.341 g). The light-yellow solution turned colorless within 20 minutes and a white precipitate formed; the reaction was monitored by HPLC-MS until starting material was consumed (ca. 1 hour). The mixture was purified via reverse phase chromatography and 0.18 mL of 1N HCl was added to the combined fractions containing product. Upon concentration, the title compound was obtained as a red tar, which solidified upon standing (70.0 mg, 69%). ^1^H NMR (400 MHz, D_2_O) δ 4.01 (ddd, J = 7.5, 6.0, 2.8 Hz, 1H), 3.87–3.77 (m, 2H), 3.76–3.71 (m, 1H), 3.71–3.64 (m, 2H), 3.56 (dd, J = 14.7, 7.5 Hz, 1H). ^13^C NMR (101 MHz, D_2_O) δ 160.9, 150.8, 150.4, 82.6, 72.2, 72.0, 70.3, 62.3, 44.5. HRMS (m/z): calculated for C_9_H_17_N_4_O_6_ ([M+H]^+^) 277.1143; found 277.1143.

### MAIT cell activation assay

Human MR1 was sub-cloned from a plasmid expressing human MR1 (Origene) into retroviral vector MSCV-IRES-GFP-R1 (MIG-R1; addgene plasmid # 27490) [41]. C1R (ATCC) cells, a human B cell lymphoblastoid cell line lacking major-histocompatibility proteins, were transduced with this construct. GFP^+^MR1^+^ cells were isolated by fluorescence activated cell sorting and cultured in IMDM media (ATCC) 10% FBS for 72 hours to confluence prior to activation. Synthetic 5-A-RU•HCl was stored at 4°C in solid form until dissolving in sterile, distilled H_2_O and freezing at –80°C in 200 μM stock solutions (referred to here as 5-A-RU•H_2_O solutions). Stock solutions of 2 μM 5-A-RU•H_2_O and 50 μM MeG•H_2_O were prepared as needed for cell culture. C1R GFP MR1 cells were directly incubated with 2 μM 5-A-RU•H_2_O and 50 μM MeG•H_2_O for 15 hours at 37°C and stained with Zombie Red Viability dye (Biolegend) and PE MR1 antibody (26.5, Biolegend) for 30 minutes at 4°C, as previously reported [24]. Following Mak et al. [37], cryopreserved healthy donor peripheral blood mononuclear cells (PBMCs) were thawed and cultured in RPMI 1640 media (ATCC) 10% FBS and directly incubated with 2 μM 5-A-RU•H_2_O/50 μM MeG•H_2_O for 15 hours at 37°C. No toxicity to C1R GFP or PBMCs was observed with 5-A-RU or MeG alone, or in combination. PBMCs were stained with Zombie Red Viability stain, Alexa 700 CD3 antibody (UCHT1, Biolegend), APC CD161 antibody (DX12, BD), and PE MR1/5-OP-RU tetramer for 30 minutes at 4°C. Cells were permeabilized and fixed for 40 minutes at 4°C with FoxP3/Transcription factor Staining Buffer Set (eBioscience) and stained with FITC granzyme B (GB11, Biolegend) for 1 hour at 4°C. Blocking experiments were performed by incubating PBMCs directly with 5 μg/mL anti-MR1 antibody (26.5, Biolegend) for one hour prior to incubation with 5-A-RU/MeG. Human CD3/CD28 T cell activator Dynabeads (Gibco) were incubated with PBMCs for 15 hours at a 1:2 bead to cell ratio as a positive control for T cell activation. All cells were analyzed on a Fortessa Flow Cytometer (BD).

### Tetramer formation

Production of MR1/5-OP-RU tetramers followed published methods with slight modifications [24]. We prepared an expression construct consisting of DNA coding for residues 1–280 of the mature human MR1 subunit followed by a Gly-Ser linker and the BirA substrate peptide 85 [42]; all codons were optimized for expression in *E*. *coli*, the synthetic gene was obtained from IDT (https://www.idtdna.com), and the insert was cloned into a pET derived vector developed in the NIH Tetramer Facility [43]. The MR1 subunit was expressed in BL21(DE3) cells and the inclusion bodies were washed and solubilized in freshly prepared 8M urea as described [43]. Folding of the MR1 subunit with human β2m, 5-A-RU, and MeG and subsequent purification followed published protocols [24]. Purified MR1/5-OP-RU was enzymatically biotinylated as described [43] and free biotin was removed by gel filtration chromatography. Tetramers were prepared with R-phycoerythrin (Prozyme, http://www.prozyme.com) as described [43].

## Results and discussion

### Chemistry

**Fig 2:**
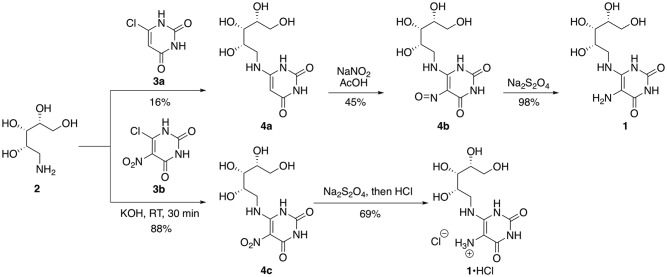
Synthetic routes to 5-A-RU 1 and its salt 1•HCl.

The synthesis of **1** requires (1) the preparation of ribitylamine, (2) attachment of ribitylamine to a uracil derivative, and (3) functional group conversions in the assembled compound to afford **1** (Fig 2). A satisfactory literature-based procedure for the conversion of (–)-ribose to ribitylamine via an intermediate oxime, which is then reduced, has been used in most reported syntheses of 5-A-RU [25, 29]. Two alternatives for the nucleophilic aromatic substitution (S_N_Ar) reaction appear in the literature. The first is a reaction of amine **2** with 6-chloropyrimidine-2,4-dione **4a**, followed by nitrosylation to afford **4b** [25]. In general, material was stored as **4b** and reduced immediately before the amine was needed. For example, amine **1** was prepared and used as a stock solution for the specific application of MR1 tetramer formation [24].

The conversion of **3a** to **4b** was originally reported to proceed in 50–70% yields for both the S_N_Ar and nitrosylation steps [25]. We briefly examined a modification whereby the original S_N_Ar conditions (neat, 95–110°C, 3 hours) were replaced by microwave conditions (180°C, 10 minutes), but in our hands the yields topped out at about 16% prior to nitrosylation. We were accordingly drawn to an alternative route initially reported by Cresswell and Wood [31], whereby the displacement was carried out on the more highly activated nitrouracil **3b** [40], which afforded **4c** smoothly in very high yield, was stable indefinitely, and could be used without additional purification.

The reduction of **4c** proceeds smoothly using either Na_2_S_2_O_4_ [31, 33] or catalytic hydrogenation [35, 44–46] to afford **1** as reported. Although these procedures been reported in acidic media [32, 35], the isolation of the unstable amine as its salt as a means of stabilizing pure material has not been previously disclosed. Since doing so would remove potential nucleophilic or single-electron-transfer proclivities from the compound, we explored salt formation of material obtained from chromatographic purification of **1**. Accordingly, addition of aqueous HCl to newly-chromatographed **1** afforded the corresponding HCl salt as a reddish solid. This material was amenable to full characterization and storage in the solid state (the NMR spectrum indicated minimal decomposition after 19 days of storage in D_2_O at room temperature without light protection or after 37 days in solid form at room temperature (wrapped in foil); see Supporting Information, S1 and S2 Figs). In comparison, we observed substantial decomposition to unknown byproducts in the NMR spectra of samples of neutral **1** kept in DMSO–*d*^6^ or D_2_O in as little as after one day of storage (Supporting Information, S3 and S4 Figs, respectively). We have prepared **1**•HCl on 200 mg scale using this approach (50% yield on this scale).

### Functional evaluation of synthetic 5-A-RU•HCl

**Fig 3:**
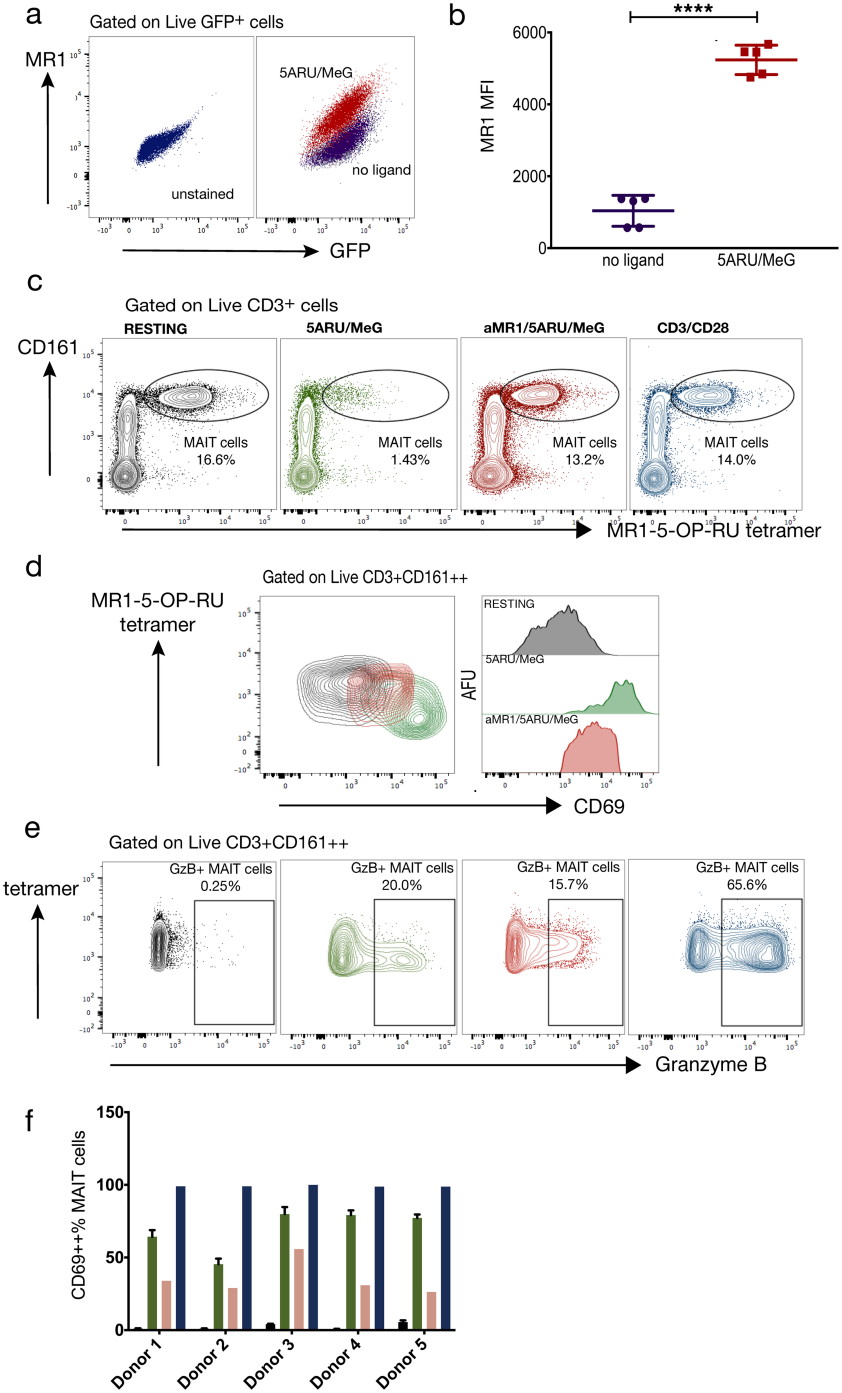
Functional studies. 5-Amino-6-(D-ribitylamino)uracil (5-A-RU) reacted with methylglyoxal (MeG) upregulates MR1 and activates human mucosal-associated invariant T (MAIT) cells. (a, b) 2 μM 5-A-RU•H_2_O and 50 μM MeG•H_2_O were directly incubated with the human C1R GFP MR1 expressing cell line for 15 hours. MR1 mean fluorescence intensity (MFI) was compared to resting C1R GFP by unpaired t-test. Graphed values are five technical replicates from two independent experiments; violet = no ligand, red = 2 μM 5ARU/50 μM MeG. **** p<0.001 (c) Human MAIT cells identified by flow cytometry as Live CD3+ MR1 tetramer+ CD161++ cells after 15 hours under the following conditions: black = no ligand, green = 2 μM 5ARU/50 μM MeG, red = αMR1/2 μM 5ARU/50 μM, blue = anti-CD3/CD28. Color code also applies to (d)-(f). (d) Contour plots and histograms representing MR1-dependent CD69 expression of MAIT cells in one human donor (e) Contour plots of MAIT cell granzyme B production under the same conditions in panel (c). (f) Mean and SD of MR1-dependent MAIT cell CD69 expression in five human donors. Mean and SD for resting and 5-A-RU/MeG conditions represent two technical replicates per condition. SD: standard deviation.

To functionally validate our synthetic 5-A-RU, we tested the compound for its ability to upregulate surface MR1 in C1R lymphoblastoid cells engineered to express human MR1, as previously reported [16]. Direct incubation with 2 μM 5-A-RU•H_2_O and 50 μM MeG•H_2_O strongly upregulated surface MR1 expression as measured by MR1 antibody staining and flow cytometry (Fig 3a and 3b).

We produced MR1/5-OP-RU tetramers from human MR1 and β2m folded with synthetic 5-A-RU•HCl and MeG using slight modifications of published protocols as described in the Methods section [24]. Fig 3c is a representative panel of human MR1/5-OP-RU tetramer staining of human peripheral blood mononuclear cells (PBMCs). The tetramer clearly identifies a distinct CD161 high cell population that comprised 16.6% of live CD3+ cells. In five healthy donors tested, the mean tetramer+CD161++% of T cells was 8.2% (Range: 3.05–16.6%). Greater than 88% of MR1 tetramer positive cells among T cells co-stained for Vα7.2 (range: 81–97%) whereas >50% of Vα7.2+ cells among T cells co-stained with tetramers (range: 21–84%) (Supporting Information, S5a Fig). Among CD3+CD161++ cells, we observed that >90% of tetramer+CD161++ T cells were identified by anti-Vα7.2 (range: 87–98%), whereas >80% of Vα7.2+CD161++ were identified by tetramers (range: 71–82%) (Supporting Information, S5 Fig). These data highlight the importance of using MR1 tetramers to identify human MAIT cells due to the presence of both Vα7.2 negative MR1-restricted cells as well as TRAV1-2 usage among naive αβ T cells [47, 48].

To determine whether synthetic 5-A-RU•HCl is able to activate MAIT cells, we employed a functional assay using human PBMCs as previously reported [37]. Although *in vitro* activation of MAIT cells was previously achieved using immortalized cell lines [16, 37, 47, 49, 50], sorted primary cells [11, 49, 50], bacteria/bacterial products [16, 21, 22, 37, 47, 49] or pan-T cell mitogen [12, 21] these assays are limited by allo-reactivity, ligand abundance/specificity, and often cannot easily be applied without additional manipulations of clinical samples. To simplify these approaches, it has been reasoned that MR1-expressing cells such as monocytes and B cells [49, 51] within the PBMC aliquot are abundant and could directly present synthetic ligand to MAIT cells without the need for culturing cell lines or performing cell-sorting [37, 52]. We directly incubated PBMCs with 2 μM 5-A-RU•H_2_O and 50 μM MeG•H_2_O and assayed upregulation of the T cell surface activation marker CD69. Synthetic 5-A-RU/MeG strongly induced MAIT TCR downregulation, accompanied by upregulation of CD69, and both responses were reversed by MR1 blockade, indicating their specificity for MR1 antigen presentation (Fig 3d). We note that Mak et al. obtained analogous activation using 1.26 nM solutions of pre-formed 5-OP-RU [37].

To confirm that TCR downregulation occurred with activation, we titrated the concentration of activation ligand and stained with tetramer or anti-Vα7.2 (Supporting Information, S5 Fig). We observed a dose-dependent loss of both surface markers, suggesting that MAIT cell activation results in TCR downregulation, as has been noted with other T cells [53]. While no toxicity was observed in PBMCs after incubation with 5-A-RU/MeG, we did observe dose-dependent depletion of MAIT cells from the tetramer positive gate, which is likely attributable to TCR downregulation and possibly activation-dependent cell death [48, 54].

We also observed upregulation of granzyme B in MAIT cells with ligand stimulation (Fig 3e). We next applied this assay to 5 independent healthy donors and observed MR1-dependent MAIT cell activation in all donors (Fig 3f). Our data confirm the functionality of the synthetic MR1 ligand precursor and establish a whole PBMC assay which can be applied to clinical samples to interrogate human MAIT cell function.

## Conclusions

These data indicate that synthetic 5-A-RU•HCl is a stable replacement of the free base in a variety of protocols. Besides improving the convenience of using of using **1** in the study of riboflavin biosynthesis [38], this synthetic method can provide abundant amounts of this MR1 ligand precursor in stable form and has facilitated construction of human and mouse MR1/5-OP-RU tetramers now available through the NIH Tetramer Core facility (http://tetramer.yerkes.emory.edu/reagents/mr1). Further, the availability of this purified MR1 ligand precursor has enabled the development of an *ex vivo* MAIT cell activation assay using human PBMCs to directly interrogate inter-individual differences in MAIT cell function in an antigen-specific, MR1-dependent manner. Overall, the development of a reproducible synthesis of **1**•HCl greatly enhances the practicality of using this ligand in the study of MAIT cell biology by increasing the stability of the reagent in a convenient solid form.

## Supporting information

S1 Fig Stability of 5-A-RU•HCl (1•HCl) in D_2_O solution. A sample of 1•HCl was dissolved in D_2_O and stored in an NMR tube without light protection. The ^1^H NMR spectra shown were collected at (a) 0 days, (b) 4 days, (c) 12 days and (d) 19 days.
(TIF)

S2 Fig Stability of 5-A-RU•HCl (1•HCl) in solid form. A portion of sample of **1•HCl** was taken at (a) 0 days, (b) 4 days, (c) 11 days and (d) 37 days, dissolved in D_2_O and analyzed by ^1^H NMR without light protection.
(TIF)

S3 Fig Stability of 5-A-RU (neutral) in DMSO-*d*^6^. (a) 0 h, (b) 21 h, (c) 5 days, and (d) 23 days. Analyzed by ^1^H NMR without light protection.
(TIF)

S4 Fig Stability of 5-A-RU (neutral) in D_2_O. (a) 0 h, (b) 21 h, (c) 5 days, and (d) 23 days. Analyzed by ^1^H NMR without light protection.
(TIF)

S5 Fig MR1/5-OP-RU tetramer and anti-Vα7.2 co-staining of T cell populations at rest and after 5ARU/MeG activation demonstrate tetramer specificity for MAIT cell identification. (a) Co-staining of resting human T cells using anti-Vα7.2 (left panel) or MR1/5-OP-RU tetramers (right panel). Results represent density plots from one donor and mean% +SD in three donors. SD: standard deviation. (b) Co-staining of resting human CD161++ T cells comparing tetramer+ cells among Vα7.2+CD161++ cells (left panel) to Vα7.2+cells among tetramer+CD161++ cells (right panel). Results represent density plots from one donor and mean% + SD in three donors. (c) Human MAIT cells were identified by flow cytometry using anti-Vα7.2 (left column) or MR1/5-OP-RU tetramers (right column) after 15 hours of rest or 5-A-RU dose titration (20 μM, 2 μM, 200 nM, 20 nM) + 50 μM MeG and demonstrate 5ARU dose-dependent TCR downregulation.
(TIF)

S1 File Additional experimental details. Includes synthesis details, copies of ^1^H and ^13^C NMR spectra of known compounds, and references for the experimental section.
(PDF)
