# Supplementary figures and images for: Synthesis, stabilization, and characterization of the MR1 ligand precursor 5-amino-6-D-ribitylaminouracil (5-A-RU)
Source: PLoS One. 2018 Feb 5;13(2):e0191837. doi: 10.1371/journal.pone.0191837 (PMC5798775; doi:10.1371/journal.pone.0191837)

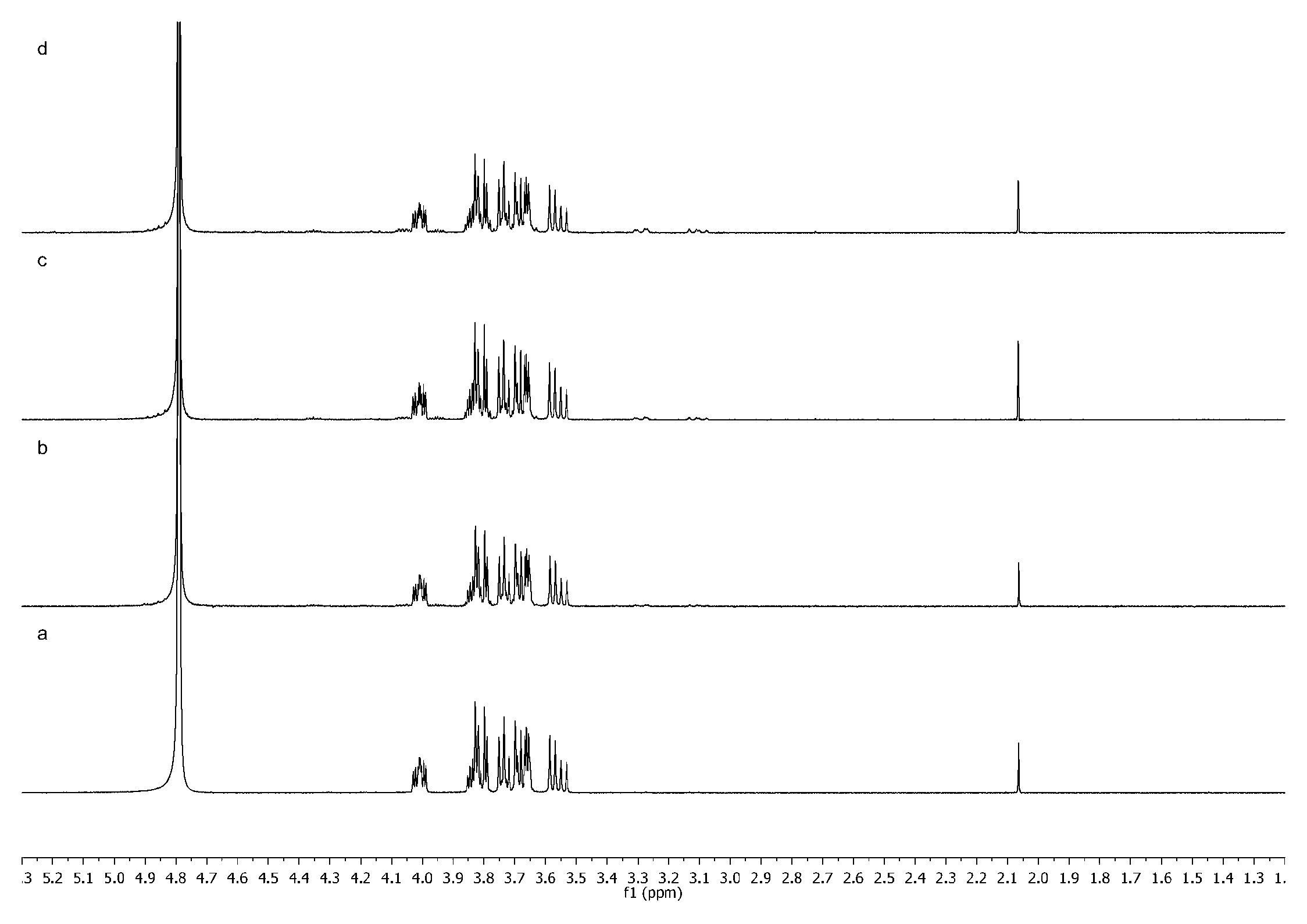

Supplement: S1 Fig — A sample of 1•HCl was dissolved in D2O and stored in an NMR tube without light protection. The 1H NMR spectra shown were collected at (a) 0 days, (b) 4 days, (c) 12 days and (d) 19 days. (TIF) [file pone.0191837.s001.tif]

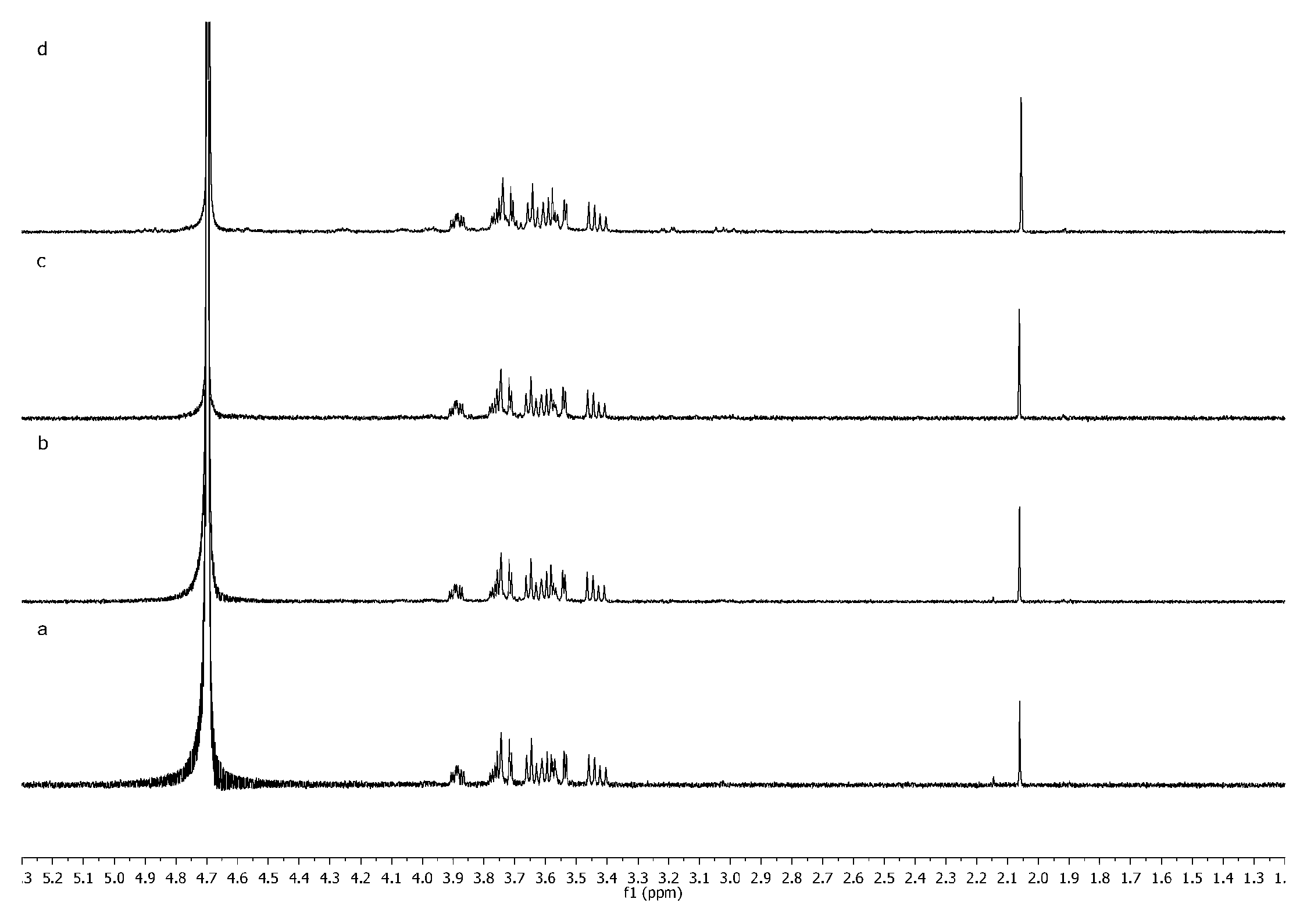

Supplement: S2 Fig — A portion of sample of 1•HCl was taken at (a) 0 days, (b) 4 days, (c) 11 days and (d) 37 days, dissolved in D2O and analyzed by 1H NMR without light protection. (TIF) [file pone.0191837.s002.tif]

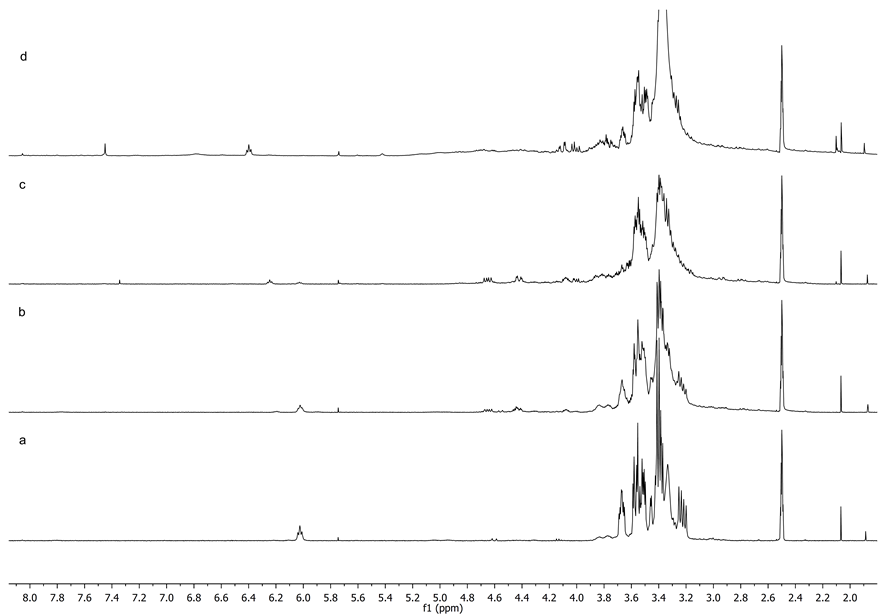

Supplement: S3 Fig — (a) 0 h, (b) 21 h, (c) 5 days, and (d) 23 days. Analyzed by 1H NMR without light protection. (TIF) [file pone.0191837.s003.tif]

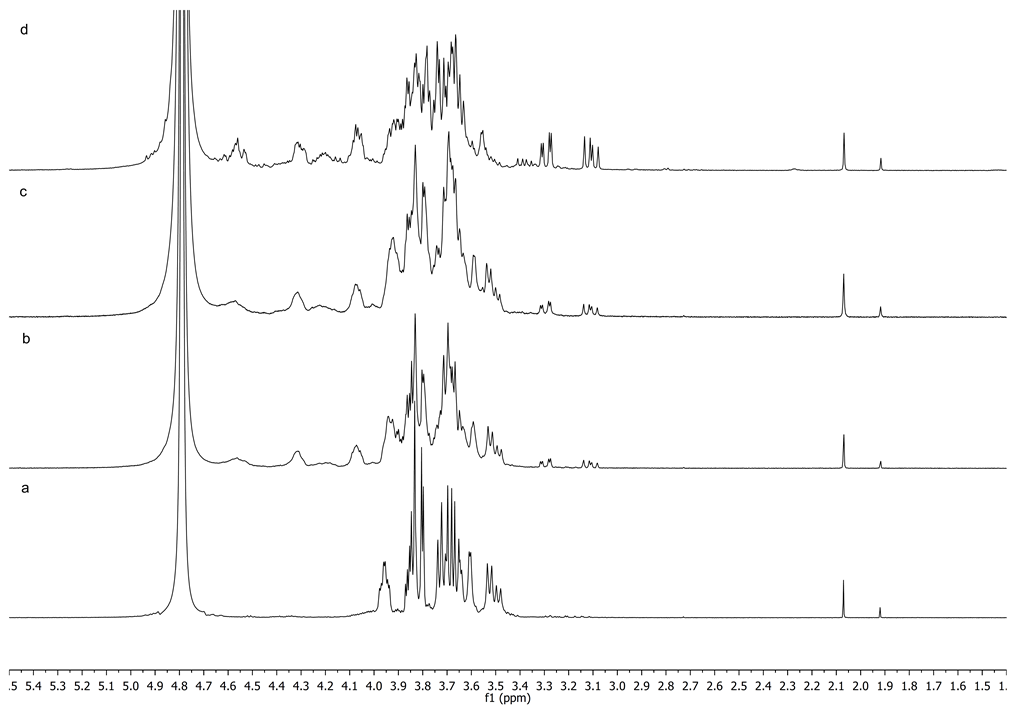

Supplement: S4 Fig — (a) 0 h, (b) 21 h, (c) 5 days, and (d) 23 days. Analyzed by 1H NMR without light protection. (TIF) [file pone.0191837.s004.tif]

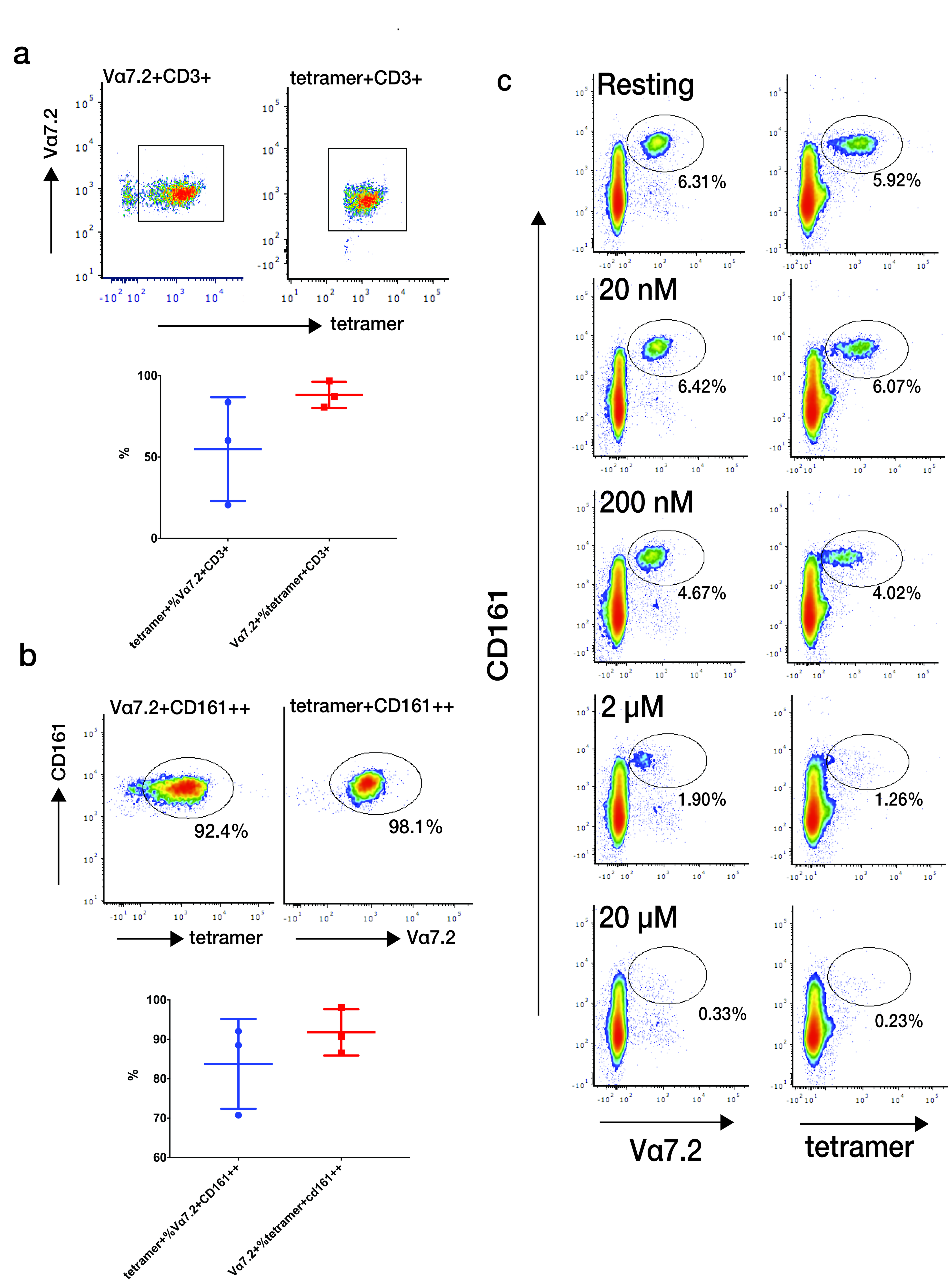

Supplement: S5 Fig — (a) Co-staining of resting human T cells using anti-Vα7.2 (left panel) or MR1/5-OP-RU tetramers (right panel). Results represent density plots from one donor and mean% +SD in three donors. SD: standard deviation. (b) Co-staining of resting human CD161++ T cells comparing tetramer+ cells among Vα7.2+CD161++ cells (left panel) to Vα7.2+cells among tetramer+CD161++ cells (right panel). Results represent density plots from one donor and mean% + SD in three donors. (c) Human MAIT cells were identified by flow cytometry using anti-Vα7.2 (left column) or MR1/5-OP-RU tetramers (right column) after 15 hours of rest or 5-A-RU dose titration (20 μM, 2 μM, 200 nM, 20 nM) + 50 μM MeG and demonstrate 5ARU dose-dependent TCR downregulation. (TIF) [file pone.0191837.s005.tif]
