# Supplementary material for: Synthesis, stabilization, and characterization of the MR1 ligand precursor 5-amino-6-D-ribitylaminouracil (5-A-RU)
Source: PLoS One. 2018 Feb 5;13(2):e0191837. doi: 10.1371/journal.pone.0191837 (PMC5798775; doi:10.1371/journal.pone.0191837)
Supplement: S1 File — Includes synthesis details, copies of 1H and 13C NMR spectra of known compounds, and references for the experimental section. (PDF) [file pone.0191837.s006.pdf]

Improved Synthesis, Stabilization, and Characterization of the MR1 Ligand Precursor 5-Amino-6-D-ribitylaminouracil (5-A-RU)

Kelin Li, Charles K. Vorkas, Ashutosh Chaudhry, Donielle Bell, Rick Willis, Alexander Rudensky, John D. Altman, Michael S. Glickman, and Jeffrey Aubé

Additional Experimental Details

Synthesis Details **S2**

Copies of ¹H and ¹³C NMR Spectra **S4**

References **S8**

Synthesis Details

Overview. This section includes experimental and spectroscopic details used to prepare the known compounds utilized in this study, including key spectroscopic data.

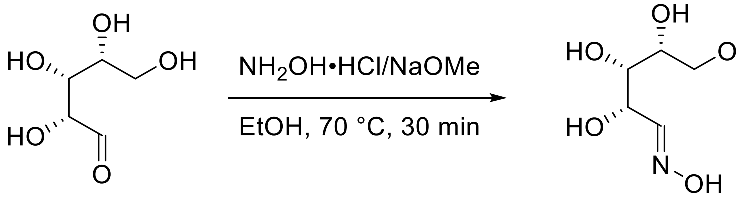

(2*S*,3*S*,4*R*)-2,3,4,5-Tetrahydroxypentanal oxime.[1] To a suspension of hydroxylamine hydrochloride (11.8 g, 170 mmol) in 60 mL of EtOH was added a solution of NaOMe (6.48 g, 120 mmol) in 60 mL of EtOH dropwise. After 5 min, the hydroxyamine solution was filtered and the filtrated was heated to 70 °C. D-(-)-Ribose (10.9 g, 72.6 mmol) was added in small portions until all the material was dissolved. The mixture was stirred at 70 °C for 30 min, then cooled to room temperature and left standing overnight. The precipitate was filtered and washed with ethanol to give (2*S*,3*S*,4*R*)-2,3,4,5-tetrahydroxypentanal oxime as a white solid (7.38 g, 61.6%). ^{1}H NMR (400 MHz, D_2O): *Z/E* isomer mixture. *Z*-isomer δ 6.94 (d, *J* = 6.3 Hz, 1H), 5.11 (dd, *J* = 6.3, 3.7 Hz, 1H), 3.92 – 3.77 (m, 3H), 3.77 – 3.60 (m, 3H). *E*-isomer δ 7.57 (d, *J* = 6.8 Hz, 1H,), 4.47 (dd, *J* = 6.8, 4.6 Hz, 0H), 3.92 – 3.77 (m, 3H), 3.77 – 3.60 (m, 3H). ^{13}C NMR (101 MHz, D_2O) *Z*-isomer δ 151.5, 72.7, 71.0, 65.3, 62.6. *E*-isomer δ 151.1, 73.0, 71.4, 69.4, 65.31, 62.5.

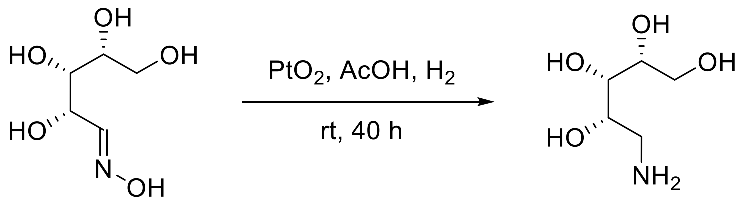

(2*R*,3*S*,4*S*)-5-Aminopentane-1,2,3,4-tetraol.[2] To a solution of (2*S*,3*S*,4*R*)-2,3,4,5-tetrahydroxypentanal oxime (3 g, 18.17 mmol) in acetic acid (35.4 mL), was added platinum(IV) oxide (0.083 g, 0.363 mmol). The mixture was purged with H_2 and stirred under a H_2 balloon for 40 h. The reaction mixture was filtered and concentrated. The residue was purified by adding to Dowex® 50WX8 hydrogen form followed by elution with water and 3*N* ammonium hydroxide. The eluent was concentrated to give a brown oil (2.7 g, 98%). ^{1}H NMR (400 MHz, D_2O) δ 3.84 – 3.66 (m, 3H), 3.66 – 3.58 (m, 2H), 2.86 (dd, *J* = 13.5, 3.1 Hz, 1H), 2.67 (dd, *J* = 13.5, 8.3 Hz, 1H). ^{13}C NMR (101 MHz, D_2O) δ 73.1, 72.5, 72.0, 62.4, 42.2.

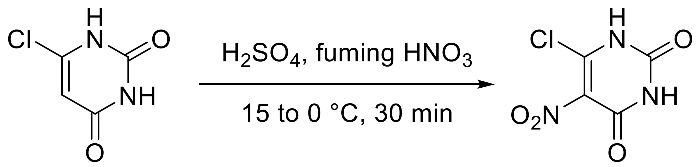

6-Chloro-5-nitropyrimidine-2,4(1*H*,3*H*)-dione. 6-Chloro-5-nitropyrimidine-2,4(1*H*,3*H*)-dione was prepared followed the procedure reported by Cheng.[3] 6-Chloropyrimidine-2,4(1*H*,3*H*)-dione (5.575 g, 38.0 mmol) was added in small portions to H_2SO_4 (18.01 mL, 338 mmol) at 15 °C. The mixture was cooled to 0 °C and 100% fuming nitric acid (6.00 mL, 134 mmol,) was added dropwise. After the addition, the reaction mixture was stirred at 10 °C for 30 min. The resulting mixture was poured into 60 g of ice (the beaker was cooled in the dry ice/acetone bath). The white precipitate was filtered and washed with ice water twice. The solid was dried under N_2 overnight to give a pale-white solid (5.52 g, 76%). The crude product was used without purification.

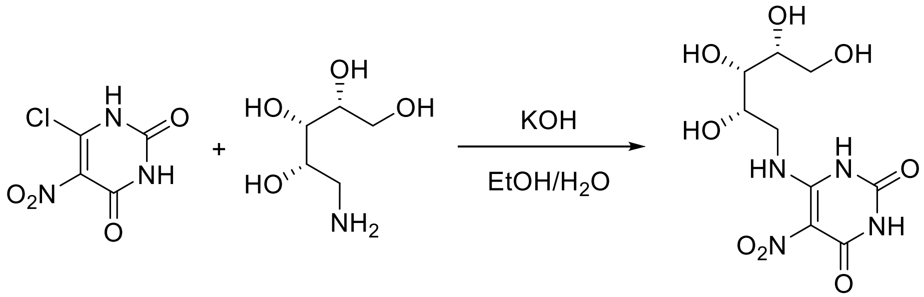

5-Nitro-6-(((2*S*,3*S*,4*R*)-2,3,4,5-tetrahydroxypentyl)amino)pyrimidine-2,4(1*H*,3*H*)-dione.[4] To a solution of (2R,3S,4S)-5-aminopentane-1,2,3,4-tetraol (2.048 mL, 5.22 mmol) in water (3.06 mL), was added 6-chloro-5-nitropyrimidine-2,4(1H,3H)-dione (0.5 g, 2.61 mmol,) in EtOH (8.08 mL). KOH (2*N*, 2.61 mL, 5.22 mmol) was added dropwise until all the starting material was consumed (monitored by LC-MS). The precipitate was filtered and washed with EtOH twice to give a light-yellow solid (0.70 g, 88%). The crude material was used directly without purification. A small portion of the product was purified via reverse phase combiflash for analysis. ^{1}H NMR (400 MHz, D_2O) δ 4.02 (td, *J* = 6.7, 3.4 Hz, 1H), 3.92 – 3.79 (m, 3H), 3.72 – 3.62 (m, 3H). ^{13}C NMR (101 MHz, D_2O) δ 161.9, 160.6, 157.7, 111.1, 72.6, 72.1, 70.2, 62.4, 43.2.

Proton and Carbon NMR spectra for previously reported compounds appear following this section.

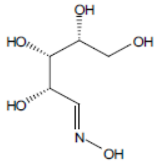
OH
HO
OH
HO
N
OH

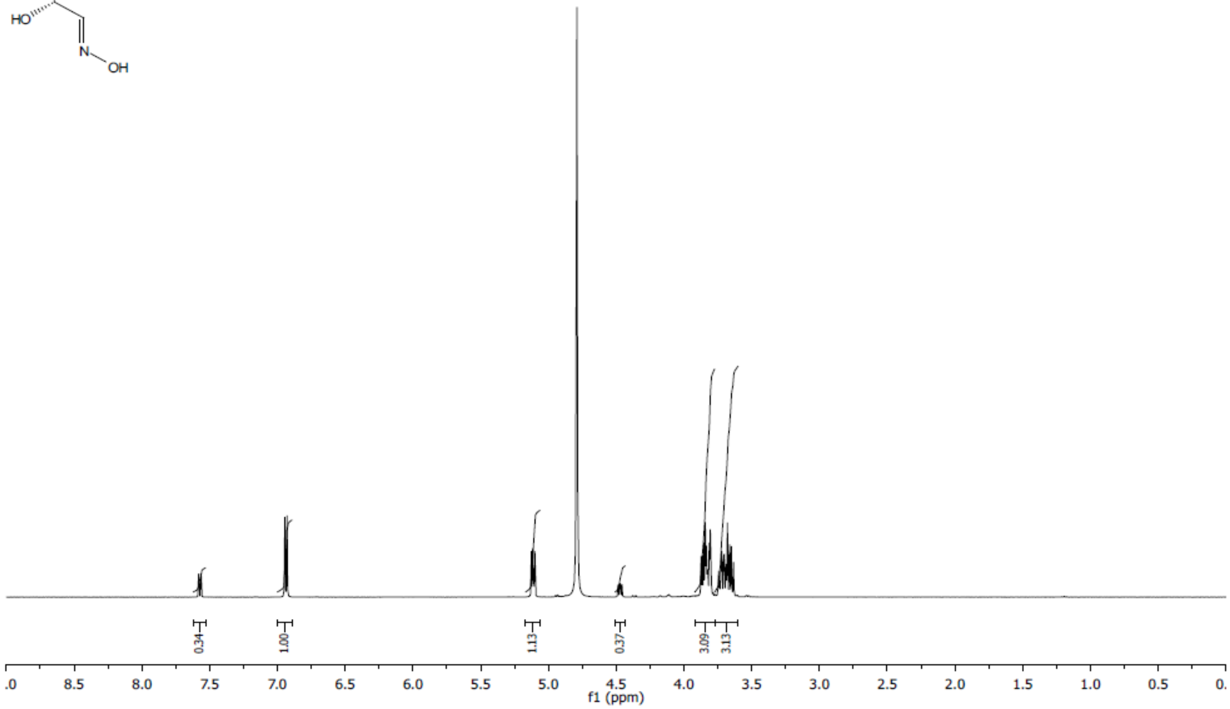
0.34
1.00
1.13
0.37
3.09
3.13
f1 (ppm)

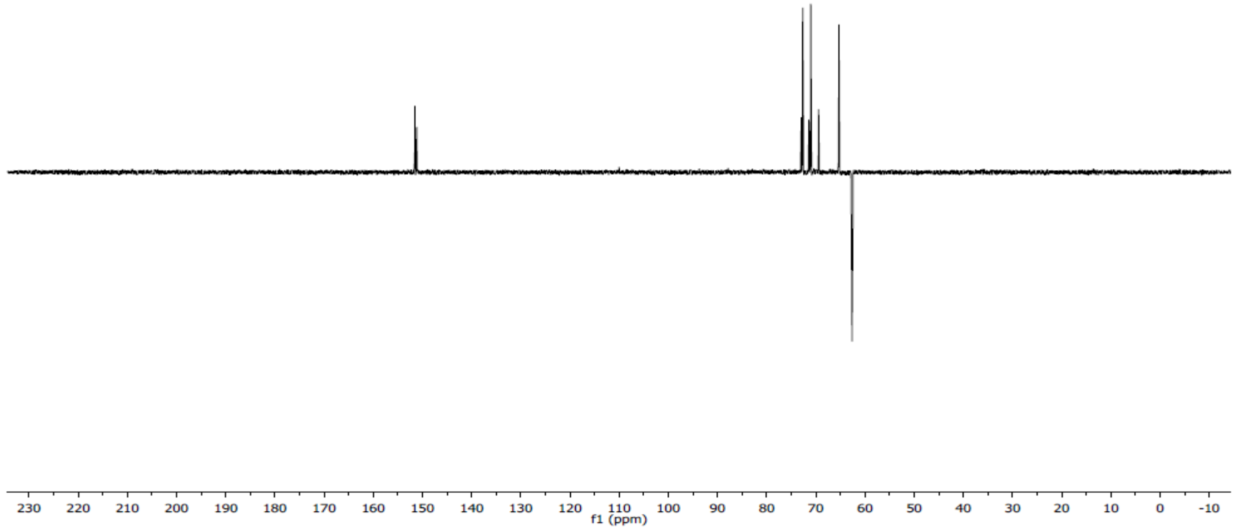
f1 (ppm)

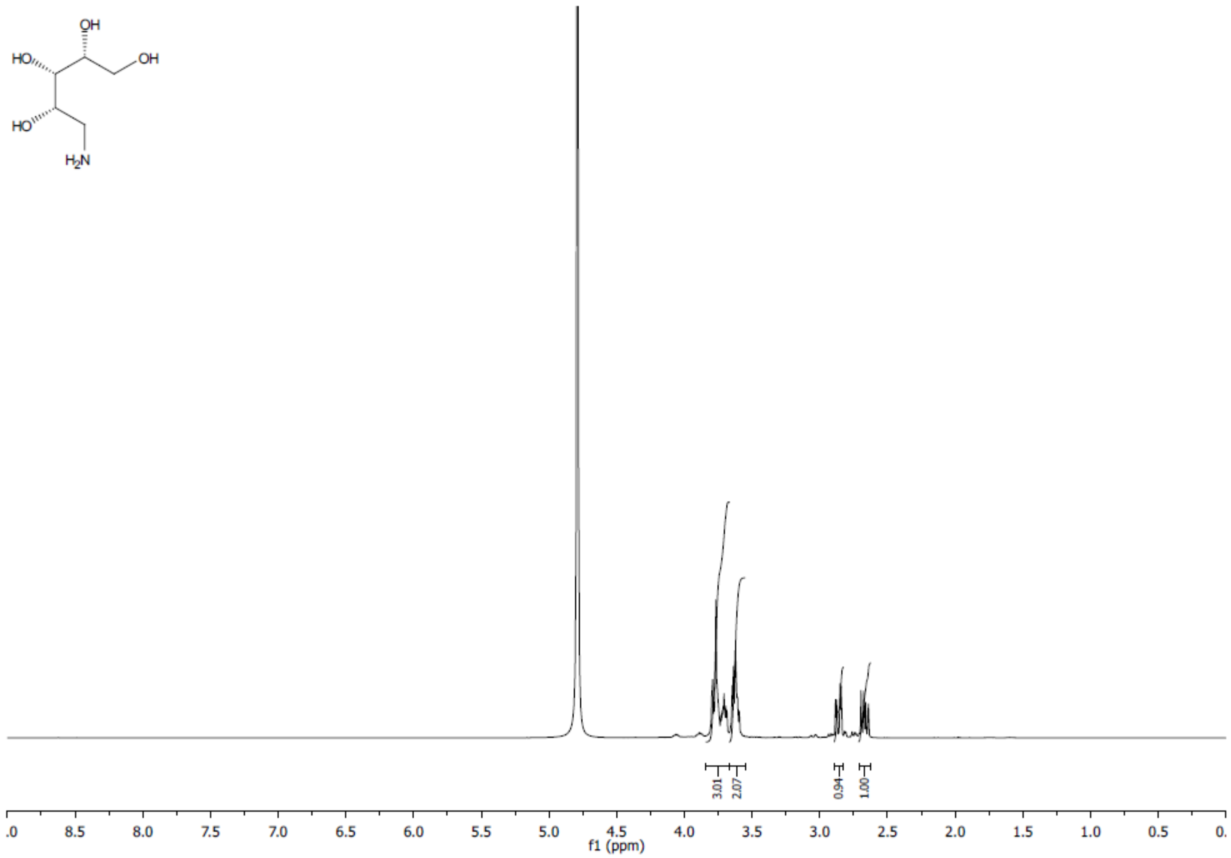
OH
HO
OH
HO
H2N
3.01
2.07
0.94
1.00
.0 8.5 8.0 7.5 7.0 6.5 6.0 5.5 5.0 4.5 4.0 3.5 3.0 2.5 2.0 1.5 1.0 0.5 0.
f1 (ppm)

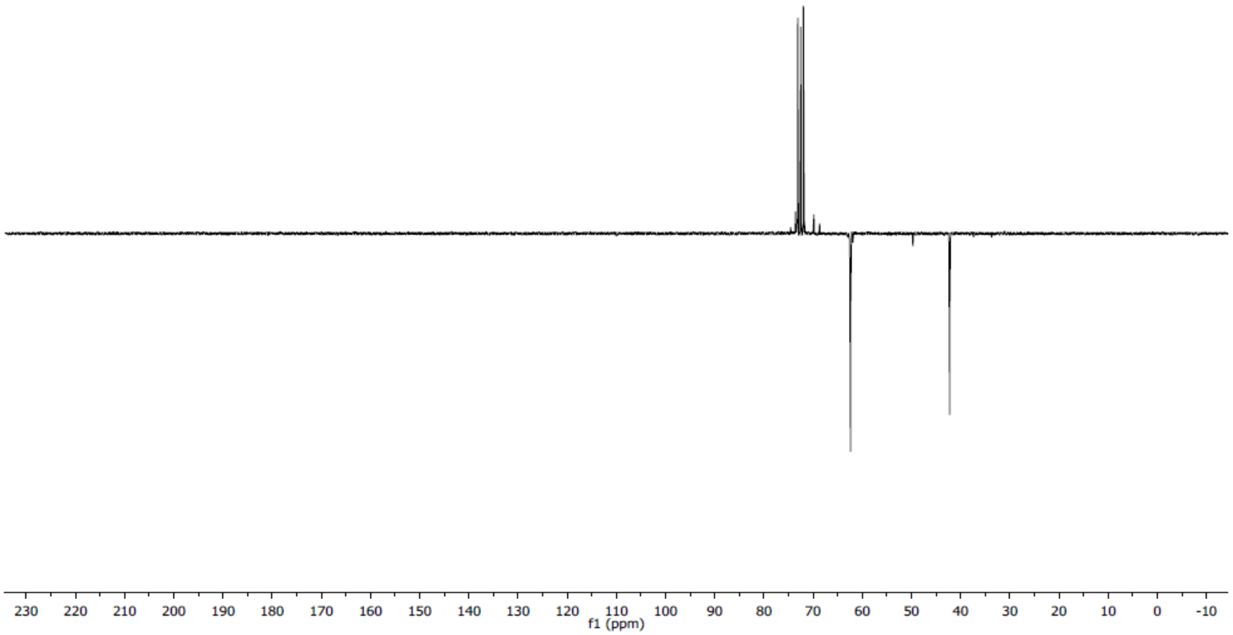
230 220 210 200 190 180 170 160 150 140 130 120 110 100 90 80 70 60 50 40 30 20 10 0 -10
f1 (ppm)

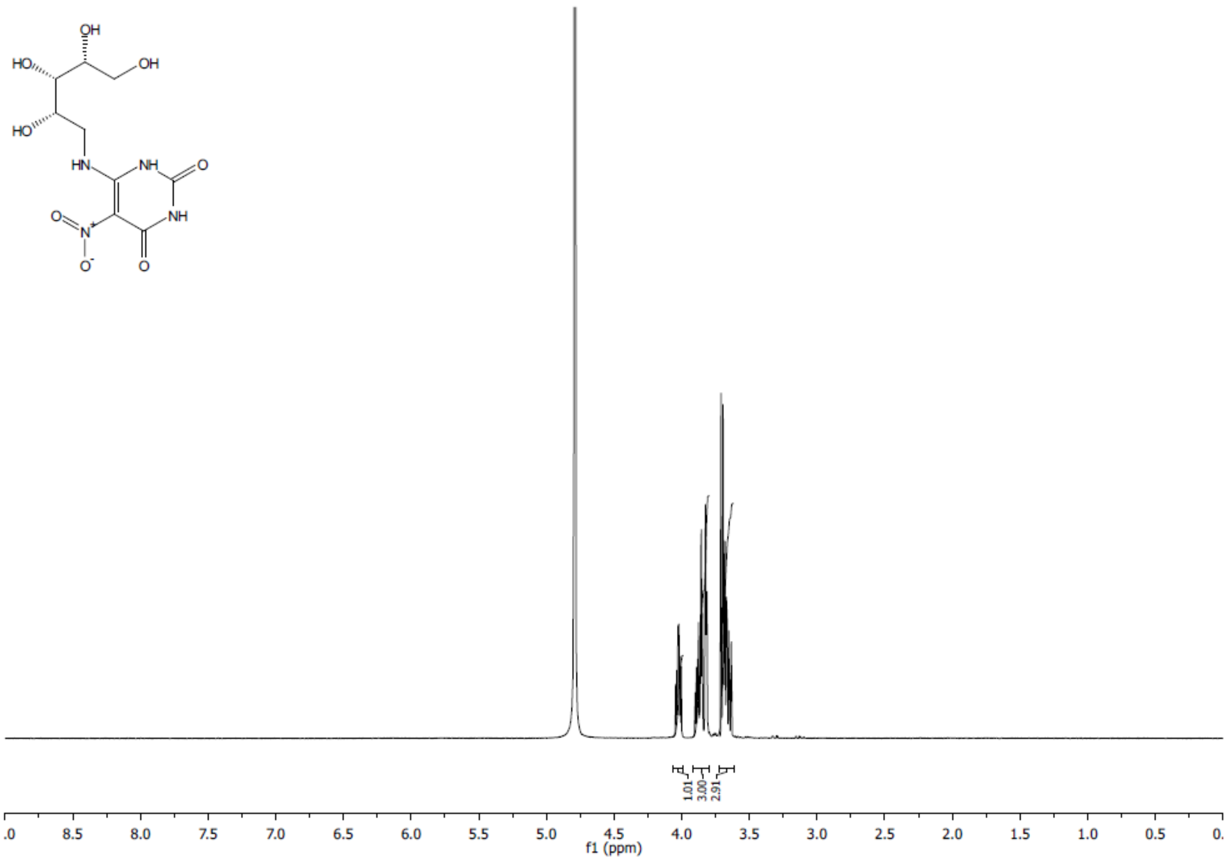
OH
HO
OH
HO
HN
NH
O
NH
O
O
N+
O-
1.01
3.00
2.91
.0 8.5 8.0 7.5 7.0 6.5 6.0 5.5 5.0 4.5 4.0 3.5 3.0 2.5 2.0 1.5 1.0 0.5 0.
f1 (ppm)

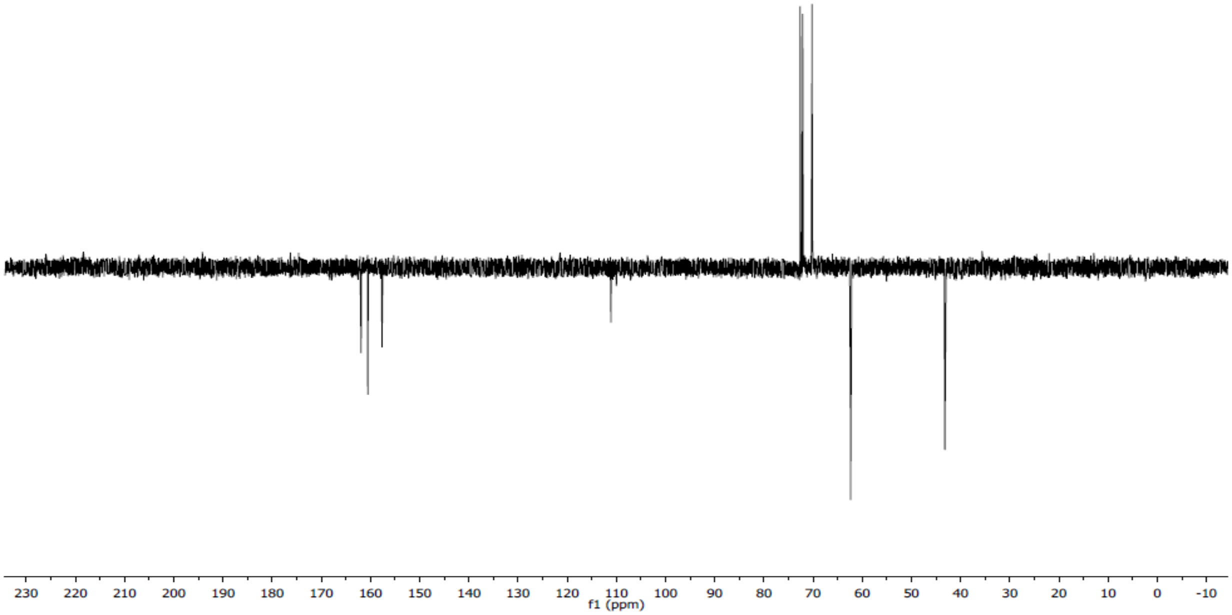
230 220 210 200 190 180 170 160 150 140 130 120 110 100 90 80 70 60 50 40 30 20 10 0 -10
f1 (ppm)

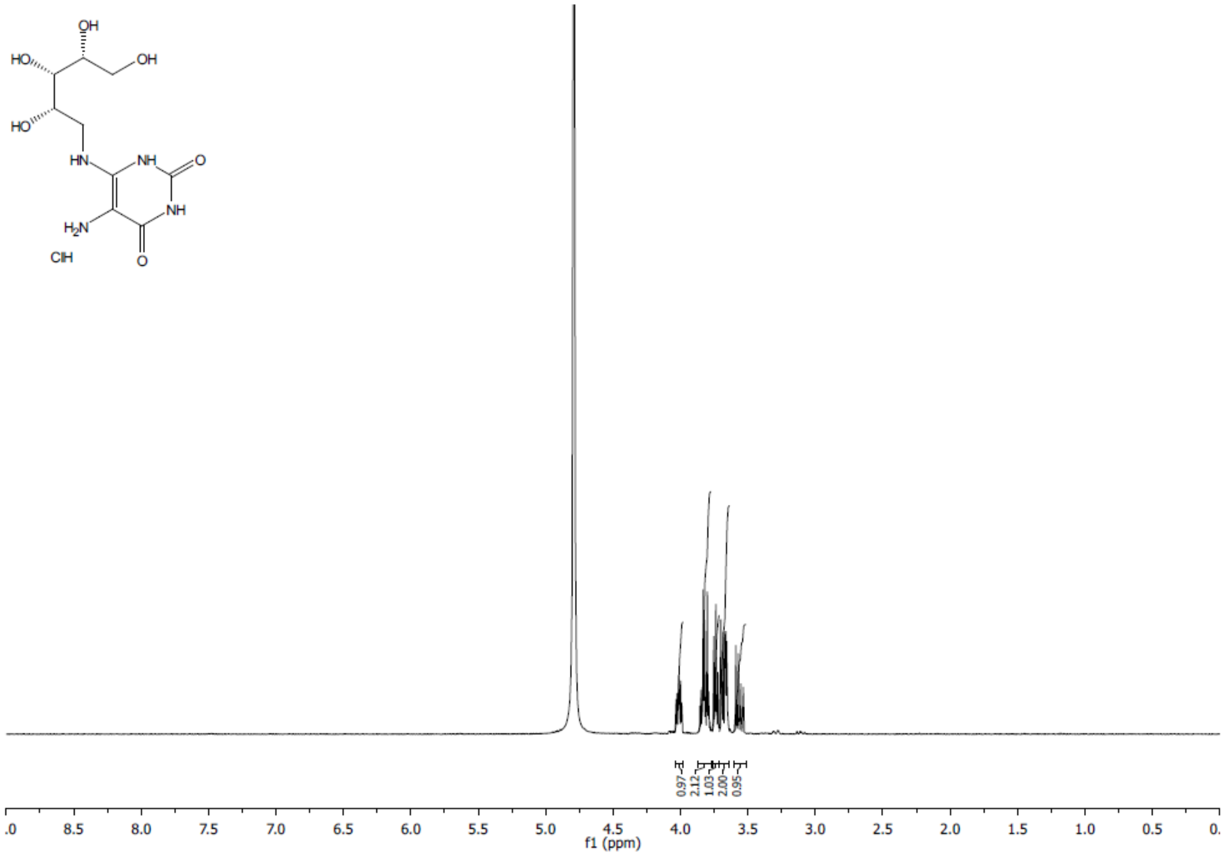
OH
HO
OH
HO
HN
NH
O
NH
H2N
ClH
O
0.97
2.12
1.03
2.00
0.95
f1 (ppm)

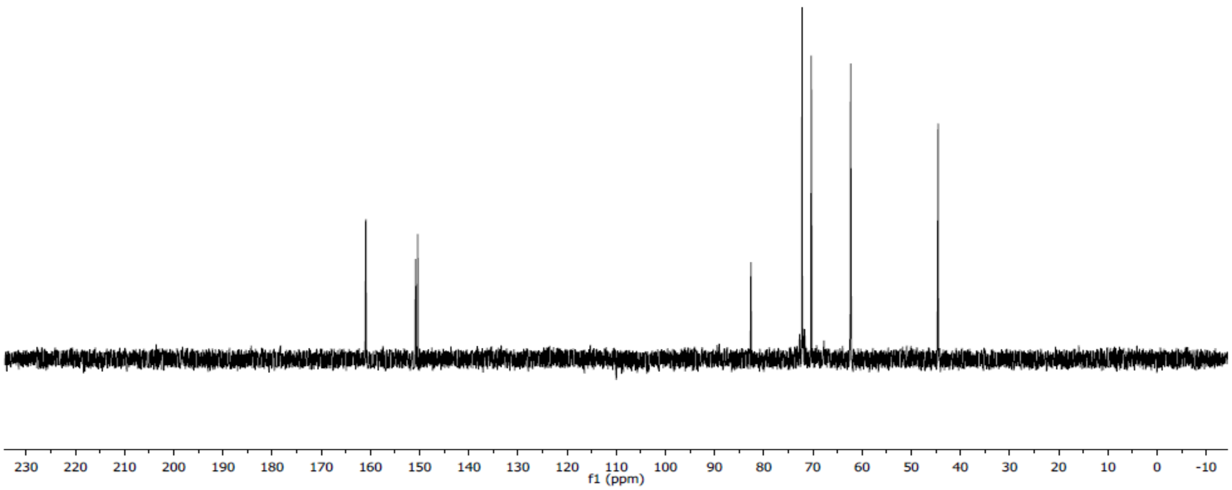
f1 (ppm)

References:

(1) Winestock, C. H.; Plaut, G. W. E., Synthesis and Properties of Certain Substituted Lumazines. *J. Org. Chem.* **1961,** *26*, 4456-4462.

(2) Maley, G. F.; Plaut, G. W. E., Isolation, Synthesis, and Metabolic Properties of 6,7-Dimethyl-8-ribityllumazine. *J. Biol. Chem.* **1959,** *234*, 641-647.

(3) Zee-Cheng, K. Y.; Cheng, C. C., Synthesis of 5,7-dioxo-3-methyl-5,6,7,8-tetrahydropyrimido[5,4-e]-as-triazine. *J. Med. Chem.* **1968,** *11*, 1107-1108.

(4) Nielsen, P.; Bacher, A. Biosynthesis of riboflavin. A simple synthesis of the substrate and product of the pyrimidine deaminase and of structural analogs. *Zeit. für Naturforsch. B* **1988**, *43b*, 1358–1364.
